# Safety and effectiveness of apremilast in Japanese patients with psoriatic disease: Results of a post‐marketing surveillance study

**DOI:** 10.1111/1346-8138.17270

**Published:** 2024-05-22

**Authors:** Mamitaro Ohtsuki, Yukari Okubo, Hidehisa Saeki, Atsuyuki Igarashi, Shinichi Imafuku, Masatoshi Abe, Siddharth Chaudhari, Masafumi Yaguchi, Ayumi Emoto, Akimichi Morita

**Affiliations:** ^1^ Department of Dermatology Jichi Medical University Shimotsuke Japan; ^2^ Department of Dermatology Tokyo Medical University Tokyo Japan; ^3^ Department of Dermatology Nippon Medical School Tokyo Japan; ^4^ Igarashi Dermatology Higashi‐Gotanda Clinic Tokyo Japan; ^5^ Department of Dermatology Fukuoka University Fukuoka Japan; ^6^ Kojinkai Sapporo Skin Clinic Sapporo Japan; ^7^ Amgen K.K. Tokyo Japan; ^8^ Department of Geriatric and Environmental Dermatology Nagoya City University Graduate School of Medical Sciences Aichi Japan

**Keywords:** apremilast, plaque psoriasis, post‐marketing surveillance, psoriatic arthritis, safety

## Abstract

The safety and efficacy of apremilast in psoriatic disease has been demonstrated in clinical trials, including in Japanese patients. This post‐marketing surveillance study was conducted after approval of apremalast in Japan in 2016 to evaluate the safety and effectiveness of the drug in Japanese patients with plaque psoriasis (PsO) and psoriatic arthritis (PsA) in routine clinical practice. Patients (enrolled between September 1, 2017, and August 31, 2019), were observed for 12 months after apremilast treatment initiation or until discontinuation or withdrawal. Safety was assessed by evaluating adverse reactions (ARs) and serious ARs. Effectiveness measures in PsO included the proportion of patients who achieved global improvement and Physician's Global Assessment (PGA) scores of 0/1 and the change from baseline in the Dermatology Life Quality Index (DLQI) after 6 and 12 months treatment. The safety analysis set included 1063 patients (PsO, *n* = 992; PsA, *n* = 127). ARs and serious ARs were reported in 29.4% and 0.7% of patients, respectively; most occurred <1 month after apremilast initiation. There were no reports of fatal ARs, serious infections, hypersensitivity, or vasculitis. No new safety signals were identified. Among the key survey items, gastrointestinal disorders were the most common ARs (21.3%). In patients with PsO, after 6 and 12 months of treatment, effectiveness rates of achieving highly effective or effective global improvement of were 90.9% and 93.8%; PGA 0/1 was achieved by 42.7% and 58.1% of patients; mean decrease from baseline in total DLQI score was 4.2 (*p* < 0.0001) and 5.7 (*p* < 0.0001), respectively. Effectiveness was evaluated in a small number of patients with PsA for some measures; after 6 and 12 months of treatment, improvements were observed in global improvement effectiveness rates, Disease Activity Score in 28 Joints score, Visual Analog Scale score, and DLQI score. We conclude that orally administered apremilast was well tolerated and effective in Japanese patients with PsO and/or PsA enrolled in this post‐marketing surveillance study.

## 1 INTRODUCTION

Plaque psoriasis (or psoriasis vulgaris; PsO) and psoriatic arthritis (PsA) are complex, multifactorial, chronic immune‐mediated inflammatory diseases that primarily affect the skin and joints respectively and are associated with a high disease burden and significantly impaired health‐related quality of life (HRQoL).^1^, ^2^

One of the key inflammatory pathways in PsO pathogenesis involves the phosphodiesterase‐4 (PDE4)‐mediated degradation of cyclic adenosine monophosphate (cAMP), a key intracellular second messenger, which promotes an increase in pro‐inflammatory mediators and a decrease in anti‐inflammatory mediators.^3^

Apremilast (Otezla®; Amgen Inc.) is an orally administered, small molecule that selectively inhibits PDE4, resulting in increased intracellular cAMP levels and subsequent downstream effects on intracellular pathways involved in innate and adaptive immunity as well as in non‐immune cells.^1^ These effects suppress the production of a broad range of pro‐inflammatory cytokines, such as tumor necrosis factor‐α, interleukin (IL)‐17, and IL‐23 and promote the expression of anti‐inflammatory cytokines such as IL‐10.^3^ As apremilast acts early in the inflammatory cascade, it is thought to lead to a broader and more balanced regulation of inflammatory mediators than biological agents that target a specific mediator.^1^

Apremilast has been studied extensively in clinical trials of patients with PsO and PsA including the phase 2b PSOR‐005 study^4^ and phase 3 ESTEEM 1 and 2 studies,^5^, ^6^ which showed that apremilast reduced the severity and extent of moderate to severe plaque psoriasis and was well tolerated. Furthermore, the phase 3 PALACE 1–4 studies^7^, ^8^, ^9^, ^10^ demonstrated the clinical benefits and favorable safety profile of apremilast among patients with PsA. In a phase 2b study conducted in Japan,^11^ apremilast demonstrated similar efficacy and tolerability to the phase 2 and phase 3 studies conducted in multiple countries,^4^, ^5^, ^6^, ^12^ confirming that data from the pivotal phase 3 studies can be extrapolated to Japanese patients. Based on those studies, apremilast was approved in Japan in 2016 for the treatment of PsO in patients with an inadequate response to topical therapies as well as for the treatment of PsA.^13^ This post‐marketing surveillance (PMS) study was conducted to investigate the safety and effectiveness of apremilast in Japanese patients with PsO and PsA treated in real‐world clinical practice.

## 2 METHODS

### 2.1 Study design

This was a multicenter, prospective, observational study (ClinicalTrials.gov identifier: NCT03284879; European Union electronic Register of Post‐Authorization Safety Studies [EU PAS] identifier: EUPAS36684) conducted in Japan in patients with PsO and/or PsA who were treated with apremilast in routine clinical practice. A total of 1086 patients were enrolled at 160 hospitals or clinics in Japan between September 1, 2017, and August 31, 2019, and were observed for 12 months after the start of apremilast treatment or until discontinuation or withdrawal. In cases of treatment discontinuation, data were recorded on concomitant medications and adverse events (AEs) for 2 months from the date of discontinuation. A centralized, electronic data capture system was used for patient registration (within 14 days of apremilast initiation) and data collection. Participating physicians completed two case report forms (CRFs) during the observation period (one after 6 months [CRF1] and the other after 12 months [CRF2] of treatment) detailing patient demographic data and baseline characteristics, apremilast treatment data, concomitant medications, AEs, disease activity scores, and patient‐ and physician‐reported outcomes.

The study was conducted in compliance with the regulatory requirements stipulated in the Good Post‐marketing Study Practice (GPSP) guidelines of Japan. Institutional review board and ethics committee approvals as well as informed consent were not required according to the GPSP ordinance. However, approval for the study was obtained if requested by a participating medical institution.

### 2.2 Patient selection and treatment

Patients who received apremilast for the first time for PsO after an inadequate response to topical therapies and/or for PsA were enrolled in the study. Patients with a history of hypersensitivity to apremilast or to any formulation excipient(s) were excluded.

Patients initially received apremilast at a dose of 10, 20, or 30 mg, in accordance with Japanese prescribing information.^12^

### 2.3 Outcome measures

Safety was evaluated by recording all ARs and serious ARs. AEs reported by physicians other than those assessed as “not related” by physicians (including unknown/not specified) were tabulated as ARs. Serious ARs were defined as ARs assessed as “serious” by physicians. ARs were classified using preferred terms and system organ classes (SOC) in the Japanese version of the Medical Dictionary for Regulatory Activities (MedDRA/J) version 25.0. Listed/unlisted ARs were assessed based on the description in apremilast prescribing information (revised July 2020, 2nd edition). In this survey, safety information was collected with “gastrointestinal disorders,” “serious infections,” “serious hypersensitivity,” “weight decreased,” “vasculitis,” “malignancies” and “depression and suicidal events” as the key survey items. The occurrence of ARs by time, number and the percentage of patients who experienced ARs, especially gastrointestinal disorders, in each of the patient background categories were recorded to identify factors potentially affecting the safety of apremilast.

Treatment effectiveness in patients with PsO was evaluated by the effectiveness rate, which was the proportion of patients who reported a global improvement of highly effective or effective, Physician's Global Assessment (PGA) scores of 0/1, DLQI score 0/1, or <5 and the change from baseline in the patient‐reported DLQI, after 6 and 12 months of treatment.^14^, ^15^ Effectiveness in patients with PsA was assessed by determining the proportion of patients who achieved global improvement and change from baseline in the Visual Analog Scale (VAS) scores for pain (0–100 mm, assessed by patients), Disease Activity Score in 28 Joints (DAS28‐CRP), and DLQI after 6 and 12 months of treatment.^16^

### 2.4 Statistical analysis

The target population size was 1000 patients. Based on the results of a Japanese clinical trial^11^ in which three of 241 (1.2%) patients with PsO experienced serious infections, the threshold of serious infections in this study was set at 1.2%. It was estimated that 814 patients would need to be included in the safety analysis set to have ≥80% power for detecting serious infections at the specified threshold (1.2%), even if the true risk was more than twice this threshold (i.e., >2.4%). Therefore, the planned population size was set at 1000 patients to account for dropouts during the 12‐month observation period.

The safety analysis set included all patients with completed CRFs who did not meet safety exclusion criteria (i.e., registration outside the contract period, enrollment after 14 days of apremilast administration, patient duplication, no apremilast administration, treatment initiation prior to 1 September 2017, and no hospital visit after apremilast initiation). The effectiveness analysis set included all patients in the safety analysis set who did not meet the following effectiveness exclusion criteria: treatment of diseases other than approved indications, previous apremilast treatment, no effectiveness assessments, and patients not fulfilling “inadequate response to topical therapies” criteria.

Data were summarized using descriptive statistics, with means and standard deviations (SD) and medians and ranges (minimum and maximum) used for continuous variables, and the number and proportion of patients used for categorical variables. The relationship between patient baseline characteristics and safety was evaluated using the Fisher's exact or Pearson's chi‐squared tests. A logistic regression Cox analysis was performed with specific items as explanatory variables (i.e., sex, age, diagnosis, disease duration, and comorbidities) to investigate risk factors for the occurrence of gastrointestinal disorders; odds ratios (ORs) and their 95% confidence intervals (CIs) were calculated. A *p*‐value of <0.05 was considered statistically significant. No imputation was made for missing data. All statistical analyses were performed using the SAS software version 9.4 (TS1M5) (SAS Institute Inc).

## 3 RESULTS

### 3.1 Patient population

At the data cut‐off (August 15, 2022), 1080 of 1086 enrolled patients had completed CRFs (Figure S1).

Patients were counted once for each diagnosis (PsO or PsA) if they had both PsO and PsA. The safety analysis set included 1063 patients, with 992 patients having PsO and 127 having PsA, meaning that 56 patients were diagnosed as having both PsO and PsA. Seventeen patients were excluded from the safety analysis set for the following reasons: enrollment after 14 days of apremilast administration (*n* = 4), no apremilast administration (*n* = 2), and no hospital visit after apremilast initiation (*n* = 11). The effectiveness analysis set included 1013 patients (954 patients with PsO and 115 with PsA); 50 patients were excluded because of no effectiveness assessments.

**FIGURE 1:**
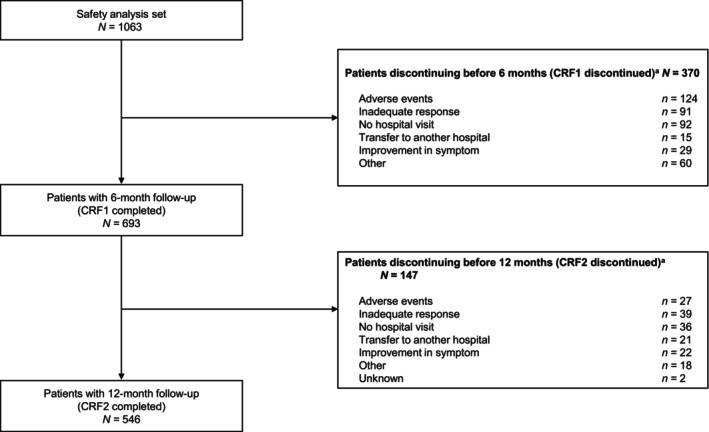
Completion and discontinuation status after 6 and 12 months of treatment. ^a^Patients were counted once for each reason if they had more than one reason for discontinuation. CRF, case report form.

In the safety analysis set, 693 of 1063 patients were followed for 6 months and completed CRF1; 370 patients had discontinued treatment, mainly due to AEs (*n* = 124), no hospital visit (*n* = 92), or inadequate response (*n* = 91; Figure 1). After 12 months' follow‐up, CRF2 was completed by 546 patients; 145 patients discontinued treatment, mostly because of an inadequate response (*n* = 39), no hospital visit (*n* = 36), or AEs (*n* = 27). The reason for discontinuation of two patients was unknown. Baseline characteristics and treatment patterns of the safety analysis set are summarized in Table 1.

**TABLE 1:** Baseline characteristics and treatment patterns of patients treated with apremilast (safety analysis set).

	Overall, *N* = 1063	PsO, *N* = 992	PsA^a^, *N* = 127
Sex, *n* (%)			
Male	745 (70.1)	698 (70.4)	82 (64.4)
Female	318 (29.9)	294 (29.6)	45 (35.4)
Age category, *n* (%), years			
<15	1 (0.1)	1 (0.1)	0
≥15 to <65	629 (59.2)	578 (58.3)	90 (70.9)
≥65	432 (40.6)	412 (41.5)	37 (29.1)
Unknown	1 (0.1)	1 (0.1)	0 (0.0)
Diagnosis			
Psoriasis vulgaris	992 (93.3)	992 (100.0)	56 (44.1)
Psoriatic arthritis	127 (11.9)	56 (5.6)	127 (100.0)
Pre‐treatment severity (PGA score) for PsO only, *n* (%)			
1 – Minor	23 (2.3)	23 (2.3)	–
2 – Mild	136 (13.7)	136 (13.7)	–
3 – Moderate	502 (50.6)	502 (50.6)	–
4 – High	177 (17.8)	177 (17.8)	–
5 – Extremely high	27 (2.7)	27 (2.7)	–
Disease duration, *n* (%), years			
<1	173 (16.3)	155 (15.6)	27 (21.3)
≥1 to <2	71 (6.7)	68 (6.9)	6 (4.7)
≥2 to <5	145 (13.6)	137 (13.8)	16 (12.6)
≥5	511 (48.1)	480 (48.4)	55 (43.3)
Unknown	163 (15.3)	152 (15.3)	23 (18.1)
Alcohol, *n* (%)	314 (29.5)	298 (30.0)	28 (22.0)
Smoking, *n* (%)	286 (26.9)	263 (26.5)	37 (29.1)
History of allergy, *n* (%)	75 (7.1)	64 (6.5)	17 (13.4)
Hospitalization, *n* (%)			
Inpatient	11 (1.0)	10 (1.0)	1 (0.8)
Outpatient	1052 (99.0)	982 (99.0)	126 (99.2)
eGFR category, *n* (%)			
≥90 mL/min/1.73 m^2^	429 (40.4)	400 (40.3)	59 (46.5)
≥60 to <89 mL/min/1.73 m^2^	292 (27.5)	256 (25.8)	53 (41.7)
≥45 to <59 mL/min/1.73 m^2^	68 (6.4)	66 (6.7)	3 (2.4)
≥30 to <44 mL/min/1.73 m^2^	18 (1.7)	16 (1.6)	6 (4.7)
≥15 to <30 mL/min/1.73 m^2^	1 (0.1)	1 (0.1)	0
<15 mL/min/1.73 m^2^	4 (0.4)	4 (0.4)	0
Past medical history^b^	260 (24.5)	235 (23.7)	43 (33.9)
Liver disorder	13 (1.2)	12 (1.2)	3 (2.4)
Kidney disorder	13 (1.2)	10 (1.0)	3 (2.4)
Psychiatric disorder	10 (0.9)	10 (1.0)	1 (0.8)
Infection	34 (3.2)	29 (2.9)	10 (7.9)
Others	196 (18.4)	182 (18.3)	28 (22.0)
Comorbidities^c^	539 (50.7)	494 (49.8)	75 (59.1)
Liver disorder	52 (4.9)	48 (4.8)	9 (7.1)
Kidney disorder	72 (6.8)	66 (6.7)	10 (7.9)
Psychiatric disorder	22 (2.1)	20 (2.0)	4 (3.1)
Infection	38 (3.6)	35 (3.5)	8 (6.3)
Others	475 (44.7)	434 (43.8)	65 (51.2)
Prior therapy			
Prior use of medications, *n* (%)	921 (86.6)	862 (86.9)	106 (83.5)
Prior use of phototherapy, *n* (%)	198 (18.6)	194 (19.6)	9 (7.1)
Targeted therapy	37 (3.5)	36 (3.6)	3 (2.4)
Narrow‐band UVB therapy	165 (15.5)	162 (16.3)	7 (5.5)
Other	5 (0.5)	5 (0.5)	0
Concomitant medications, *n* (%)	952 (89.6)	893 (90.0)	111 (87.4)
Concomitant therapy for psoriasis, *n* (%)	913 (85.9)	857 (86.4)	104 (81.9)
Immunosuppressive drugs (cyclosporine, methotrexate)	34 (3.2)	25 (2.5)	17 (13.4)
Vitamin A derivatives (retinoid)	10 (0.9)	9 (0.9)	1 (0.8)
Biologics	8 (0.8)	7 (0.7)	3 (2.4)
Topical corticosteroids	818 (77.0)	780 (78.6)	73 (57.5)
Topical vitamin D3	736 (69.2)	703 (70.9)	66 (52.0)
Phototherapy	214 (20.1)	209 (21.1)	15 (11.8)
Targeted therapy	55 (5.2)	54 (5.4)	6 (4.7)
Narrow‐band UVB therapy	171 (16.1)	167 (16.8)	10 (7.9)
Other	229 (21.5)	198 (20.0)	50 (39.4)
Concomitant therapy for diseases other than psoriasis, *n* (%)	9 (0.8)	9 (0.9)	0

Abbreviations: eGFR, estimated glomerular filtration rate; PGA, Physician Global Assessment; PsA, psoriatic arthritis; PsO, plaque psoriasis; UV, ultraviolet.

^a^ Severity of PsA is included in Table S2.

^b^ Disease or symptom that was cured before apremilast initiation.

^c^ Disease or symptom present at apremilast initiation.

Most patients were male (70.1% vs 29.9% female), aged ≥15 to <65 years (59.2% vs 40.6% aged ≥65 years and 0.1% aged <15 years; Table 1). Pre‐treatment severity (PGA score) was moderate in more than half (50.6%) of the PsO patients, and the disease duration was <1 year in 16.3% of patients, ≥1 to <2 years in 6.7%, ≥2 to <5 years in 13.6%, and ≥5 years in 48.1%. At baseline, 50.7% of patients had comorbidities, 6.8% had kidney disorder, 4.9% had liver disorder, 3.6% had infection, 2.1% had a psychiatric disorder, and 44.7% had other comorbidities. Moreover, 85.9% of patients received concomitant therapy for PsO including topical corticosteroids (77.0%), topical vitamin D3 (69.2%), phototherapy (20.1%), immunosuppressive drugs (cyclosporine, methotrexate) (3.2%), vitamin A derivatives (retinoid) (0.9%), biologics (0.8%), or other therapies (21.5%). Details related to treatment exposure are provided in Table S1.

### 3.2 Safety

Adverse reactions were reported in 312/1063 patients (29.4%) and serious ARs in 7/1063 of patients (0.7%) in the safety analysis set (Table 2). Common ARs (≥5% of patients) were diarrhea (11.7%) and nausea (5.9%). Serious ARs included colon cancer, colon cancer metastasis, hepatocellular carcinoma, cerebral infarction, myocardial infarction, hypertension, vomiting, and abnormal hepatic function (each in one patient [0.1%]). No fatal ARs were reported, and no deaths or cases of suicide overall were detected.

**TABLE 2:** Adverse reactions (ARs) in patients in the safety analysis set (*N* = 1063).

System organ class, *n* (%) preferred term	All ARs	Serious ARs
Any AR	312 (29.4)	7 (0.7)
Infections and infestations	7 (0.7)	0
Nasopharyngitis	2 (0.2)	0
Neoplasms benign, malignant and unspecified (including cysts and polyps)	2 (0.2)	2 (0.2)
Colon cancer	1 (0.1)	1 (0.1)
Colon cancer metastatic	1 (0.1)	1 (0.1)
Hepatocellular carcinoma	1 (0.1)	1 (0.1)
Metabolism and nutrition disorders	17 (1.6)	0
Decreased appetite	17 (1.6)	0
Psychiatric disorders	5 (0.5)	0
Depression	2 (0.2)	0
Nervous system disorders	36 (3.4)	1 (0.1)
Headache	24 (2.3)	0
Dizziness	6 (0.6)	0
Somnolence	2 (0.2)	0
Cerebral infarction	1 (0.1)	1 (0.1)
Ear and labyrinth disorders	3 (0.3)	0
Vertigo	3 (0.3)	0
Cardiac disorders	4 (0.4)	1 (0.1)
Myocardial infarction	1 (0.1)	1 (0.1)
Vascular disorders	3 (0.3)	1 (0.1)
Hypertension	2 (0.2)	1 (0.1)
Respiratory, thoracic and mediastinal disorders	4 (0.4)	0
Dyspnea	2 (0.2)	0
Gastrointestinal disorders	226 (21.3)	1 (0.1)
Diarrhea	124 (11.7)	0
Nausea	63 (5.9)	0
Soft feces	32 (3.0)	0
Abdominal discomfort	14 (1.3)	0
Vomiting	7 (0.7)	1 (0.1)
Dyspepsia	7 (0.7)	0
Abdominal pain	5 (0.5)	0
Abdominal pain upper	3 (0.3)	0
Frequent bowel movements	3 (0.3)	0
Gastrointestinal disorder	2 (0.2)	0
Hepatobiliary disorders	1 (0.1)	1 (0.1)
Hepatic function abnormal	1 (0.1)	1 (0.1)
Skin and subcutaneous tissue disorders	22 (2.1)	0
Worsening of Psoriasis	14 (1.3)	0
Pruritus	3 (0.3)	0
Drug eruption	2 (0.2)	0
Urticaria	2 (0.2)	0
Musculoskeletal and connective tissue disorders	10 (0.9)	0
Arthralgia^a^	5 (0.5)	0
Psoriatic arthropathy	3 (0.3)	0
Myalgia	2 (0.2)	0
General disorders and administration site conditions	21 (2.0)	0
Malaise	10 (0.9)	0
Therapeutic response decreased	4 (0.4)	0
Feeling abnormal	2 (0.2)	0
Investigations	7 (0.7)	0
Weight decreased^b^	3 (0.3)	0

*Note*: Adverse events reported by physicians other than those assessed as “not related” by physicians (including unknown/not specified) were tabulated as adverse reactions.

^a^ Causality with the drug could not be ruled out for three patients but all five patients eventually recovered or achieved remission.

^b^ Individual weight decreases were 5.4 kg, 5.5 kg, and value unknown.

The following patient baseline characteristics were associated with a significant increase in the incidence of ARs: age (≥65 years vs ≥15 to <65 years vs <15 years; *p* = 0.0014 using a chi‐squared test of independence), smoking history (*p* = 0.0284), allergy history (*p* = 0.0011), past medical history except for liver, kidney, or psychiatric disorders, or infections (*p* = 0.0091), comorbidities (*p* < 0.0001), comorbidities except for liver, kidney, or psychiatric disorders, or infections (*p* < 0.0001), concomitant medications (*p* = 0.0017; Table 3).

**TABLE 3:** Adverse reactions (ARs) by patient baseline characteristics.

	*N*	Patients with ARs, *n* (%)	*p*
Total	1063	312 (29.4)	
Sex			
Male	745	210 (28.2)	0.2115^a^
Female	318	102 (32.1)	
Age category			
<15 years	1	1 (100.0)	0.0014^b^
≥15 to <65 years	629	160 (25.4)	
≥65 years	432	150 (34.7)	
Pre‐key treatment severity (PGA score) for PsO only			
0 – None	1	0	0.3137^b^
1 – Minor	23	8 (34.8)	
2 – Mild	136	48 (35.3)	
3 – Moderate	502	146 (29.1)	
4 – High	177	45 (25.4)	
5 – Extremely high	27	5 (18.5)	
Disease duration			
<1 year	173	51 (29.5)	0.5474^b^
≥1 to <2 years	71	17 (23.9)	
≥2 to <5 years	145	48 (33.1)	
≥5 years	511	146 (28.6)	
Alcohol			
No	286	75 (26.2)	0.4131^a^
Yes	314	92 (29.3)	
Smoking			
No	361	90 (24.9)	0.0284^a^
Yes	286	94 (32.9)	
History of allergy			
No	825	219 (26.6)	0.0011^a^
Yes	75	34 (45.3)	
Hospitalization			
Inpatient	11	1 (9.1)	0.1907^a^
Outpatient	1052	311 (29.6)	
eGFR category			
≥90 mL/min/1.73 m^2^	429	107 (24.9)	0.0545^b^
≥60 to <89 mL/min/1.73 m^2^	292	99 (33.9)	
≥45 to <59 mL/min/1.73 m^2^	68	26 (38.2)	
≥30 to <44 mL/min/1.73 m^2^	18	6 (33.3)	
≥15 to <20 mL/min/1.73 m^2^	1	0	
<15 mL/min/1.73 m^2^	4	2 (50.0)	
Past medical history^c^			
No	803	225 (28.0)	0.1001^a^
Yes	260	87 (33.5)	
Liver disorder			
No	1050	306 (29.1)	0.2197^a^
Yes	13	6 (46.2)	
Kidney disorder			
No	1050	310 (29.5)	0.3663^a^
Yes	13	2 (15.4)	
Psychiatric disorder			
No	1053	310 (29.4)	0.7322^a^
Yes	10	2 (20.0)	
Infection			
No	1029	303 (29.5)	0.8488^a^
Yes	34	9 (26.5)	
Others			
No	867	239 (27.6)	0.0091^a^
Yes	196	73 (37.2)	
Comorbidities^d^			
No	524	118 (22.5)	<0.0001^a^
Yes	539	194 (36.0)	
Liver disorder			
No	1011	299 (29.6)	0.5354^a^
Yes	52	13 (25.0)	
Kidney disorder			
No	991	288 (29.1)	0.4251^a^
Yes	72	24 (33.3)	
Psychiatric disorder			
No	1041	303 (29.1)	0.2408^a^
Yes	22	9 (40.9)	
Infection			
No	1025	296 (28.9)	0.1009^a^
Yes	38	16 (42.1)	
Others			
No	588	141 (24.0)	<0.0001^a^
Yes	475	171 (36.0)	
Prior use of medications			
No	106	25 (23.6)	0.1776^a^
Yes	921	278 (30.2)	
Prior use of phototherapy			
No	839	254 (30.3)	0.3427^a^
Yes	198	53 (26.8)	
Concomitant medications			
No	108	18 (16.7)	0.0017^a^
Yes	952	293 (30.8)	
Concomitant phototherapy for psoriasis			
No	844	253 (30.0)	0.5035^a^
Yes	214	59 (27.6)	
Concomitant therapy for diseases other than psoriasis			
No	995	294 (29.6)	0.0243^a^
Yes	9	6 (66.7)	

Abbreviations: eGFR, estimated glomerular filtration rate; PGA, Physician's Global Assessment; PsO, plaque psoriasis.

^a^ Fisher's exact test.

^b^ Chi‐squared test.

^c^ Disease or symptom that was cured before apremilast initiation.

^d^ Disease or symptom present at apremilast initiation.

Most ARs were reported within 1 month of initiating apremilast treatment and decreased in number gradually over time (Table 4). Diarrhea was the only common AR (≥5% of patients) occurring within 1 month of treatment.

**TABLE 4:** Occurrence of adverse reactions (AR) by time‐period.

System organ class, *n* (%) preferred term	Duration since onset of observation					
	<1 month, *N* = 1063	≥1 to <3 months, *N* = 963	≥3 to <6 months, *N* = 835	≥6 to <9 months, *N* = 703	≥9 to <12 months, *N* = 613	≥12 months, *N* = 22
Any AR	213 (20.0)	59 (6.1)	33 (4.0)	18 (2.6)	14 (2.3)	1 (4.6)
Infections and infestations	2 (0.2)	2 (0.2)	1 (0.1)	0	1 (0.2)	0
Neoplasms benign, malignant and unspecified (including cysts and polyps)	0	0	1 (0.1)	0	1 (0.2)	0
Metabolism and nutrition disorders	8 (0.8)	7 (0.7)	1 (0.1)	1 (0.1)	0	0
Psychiatric disorders	1 (0.1)	2 (0.2)	1 (0.1)	0	0	0
Nervous system disorders	28 (2.6)	4 (0.4)	2 (0.2)	1 (0.1)	2 (0.3)	0
Ear and labyrinth disorders	2 (0.2)	0	0	1 (0.1)	0	0
Cardiac disorders	2 (0.2)	1 (0.1)	1 (0.1)	0	1 (0.2)	0
Vascular disorders	2 (0.2)	1 (0.1)	0	0	0	0
Respiratory, thoracic and mediastinal disorders	2 (0.2)	1 (0.1)	0	0	1 (0.2)	0
Gastrointestinal disorders	169 (15.9)	37 (3.8)	15 (1.8)	8 (1.1)	8 (1.3)	1 (4.6)
Hepatobiliary disorders	1 (0.1)	0	0	0	0	0
Skin and subcutaneous tissue disorders	6 (0.6)	4 (0.4)	5 (0.6)	4 (0.6)	1 (0.2)	0
Musculoskeletal and connective tissue disorders	7 (0.7)	2 (0.2)	0	1 (0.1)	0	0
General disorders and administration site conditions	12 (1.1)	4 (0.4)	4 (0.5)	1 (0.1)	0	0
Investigations	1 (0.1)	2 (0.2)	2 (0.2)	1 (0.1)	1 (0.2)	0

**FIGURE 2:**
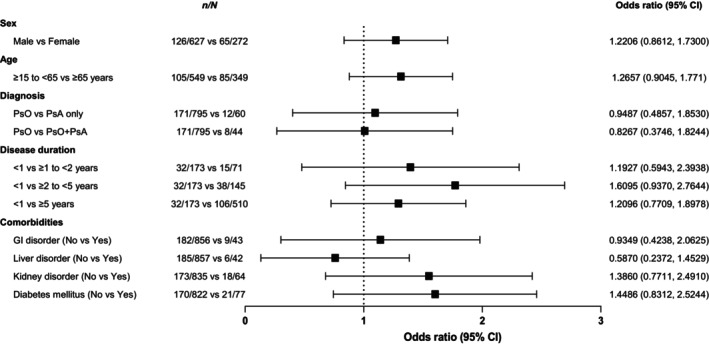
Multivariate logistic regression analyses of patient factors associated with gastrointestinal (GI) disorders. CI, confidence interval.

Among the key survey items, gastrointestinal disorders were reported in 226 (21.3%) patients, weight decreased in three (0.3%) patients, malignancies occurred in two (0.2%) patients, and depression and suicidal events in three (0.3%) patients (two cases of depression and one suicidal event but no death). Serious infections, serious hypersensitivity, or vasculitis were not reported. Baseline patient characteristics associated with a significant increase in the incidence of gastrointestinal disorders were smoking history (*p* = 0.0253), comorbidities (*p* = 0.0003), comorbidities except for liver, kidney, or psychiatric disorders, or infections (*p* = 0.0052), and prior and concomitant medications (*p* = 0.0168 and *p* = 0.0007, respectively; Table 5). In multivariate logistic regression analyses, no significant association was found between any of the explanatory variables (i.e., sex, age, diagnosis, disease duration, or comorbidities) and the occurrence of gastrointestinal disorders (Figure 2). Among the 226 patients with gastrointestinal disorders, 63 patients discontinued apremilast treatment.

**TABLE 5:** Occurrence of adverse drug reactions (ARs) of gastro intestinal disorders by patient baseline characteristics.

	*N*	Patients with ARs, *n* (%)	*p*
Total	1063	226 (21.3)	
Sex			
Male	745	153 (20.5)	0.4130^a^
Female	318	73 (23.0)	
Age category			
<15 years	1	1 (100.0)	0.0506^b^
≥15 to <65 years	629	123 (19.6)	
≥65 years	432	101 (23.4)	
Pre‐treatment severity (PGA) for PsO only			
0 – None	1	0	0.7849^b^
1 – Minor	23	7 (30.4)	
2 – Mild	136	32 (23.5)	
3 – Moderate	502	107 (21.3)	
4 – High	177	37 (20.9)	
5 – Extremely high	27	4 (14.8)	
Disease duration			
<1 year	173	32 (18.5)	0.4025^b^
≥1 to <2 years	71	15 (21.1)	
≥2 to <5 years	145	38 (26.2)	
≥5 years	511	107 (20.9)	
Alcohol			
No	286	51 (17.8)	0.1863^a^
Yes	314	70 (22.3)	
Smoking			
No	361	64 (17.7)	0.0253^a^
Yes	286	72 (25.2)	
History of allergy			
No	825	166 (20.1)	0.1357^a^
Yes	75	21 (28.0)	
Hospitalization			
Inpatient	11	1 (9.1)	0.4740^a^
Outpatient	1052	225 (21.4)	
eGFR category			
≥90 mL/min/1.73 m^2^	429	78 (18.2)	0.3099^b^
≥60 to <89 mL/min/1.73 m^2^	292	68 (23.3)	
≥45 to <59 mL/min/1.73 m^2^	68	17 (25.0)	
≥30 to <44 mL/min/1.73 m^2^	18	4 (22.2)	
≥15 to <20 mL/min/1.73 m^2^	1	0	
<15 mL/min/1.73 m^2^	4	2 (50.0)	
Past medical history^c^			
No	803	167 (20.8)	0.5417^a^
Yes	260	59 (22.7)	
Liver disorder			
No	1050	222 (21.1)	0.4915^a^
Yes	13	4 (30.8)	
Kidney disorder			
No	1050	225 (21.4)	0.3209^a^
Yes	13	1 (7.7)	
Psychiatric disorder			
No	1053	224 (21.3)	1.0000^a^
Yes	10	2 (20.0)	
Infection			
No	1029	217 (21.1)	0.5215^a^
Yes	34	9 (26.5)	
Others			
No	867	176 (20.3)	0.1217^a^
Yes	196	50 (25.5)	
Comorbidities^d^			
No	524	87 (16.6)	0.0003^a^
Yes	539	139 (25.8)	
Liver disorder			
No	1011	218 (21.6)	0.3843^a^
Yes	52	8 (15.4)	
Kidney disorder			
No	991	207 (20.9)	0.2958^a^
Yes	72	19 (26.4)	
Psychiatric disorder			
No	1041	220 (21.1)	0.4397^a^
Yes	22	6 (27.3)	
Infection			
No	1025	213 (20.8)	0.0663^a^
Yes	38	13 (34.2)	
Others			
No	588	106 (18.0)	0.0052^a^
Yes	475	120 (25.3)	
Prior use of medications			
No	106	13 (12.3)	0.0168^a^
Yes	921	205 (22.3)	
Prior use of phototherapy			
No	839	181 (21.6)	0.7008^a^
Yes	198	40 (20.2)	
Concomitant medications			
No	108	10 (9.3)	0.0007^a^
Yes	952	215 (22.6)	
Concomitant phototherapy for psoriasis			
No	844	182 (21.6)	0.7802^a^
Yes	214	44 (20.6)	
Concomitant therapy for diseases other than psoriasis			
No	995	213 (21.4)	0.1075^a^
Yes	9	4 (44.4)	

Abbreviations: eGFR, estimated glomerular filtration rate; PGA, Physician's Global Assessment; PsO, plaque psoriasis.

^a^ Fisher's exact test.

^b^ Chi‐squared test.

^c^ Disease or symptom that was cured before apremilast initiation.

^d^ Disease or symptom present at apremilast initiation.

### 3.3 Effectiveness

#### 3.3.1 Patients with PsO

**FIGURE 3:**
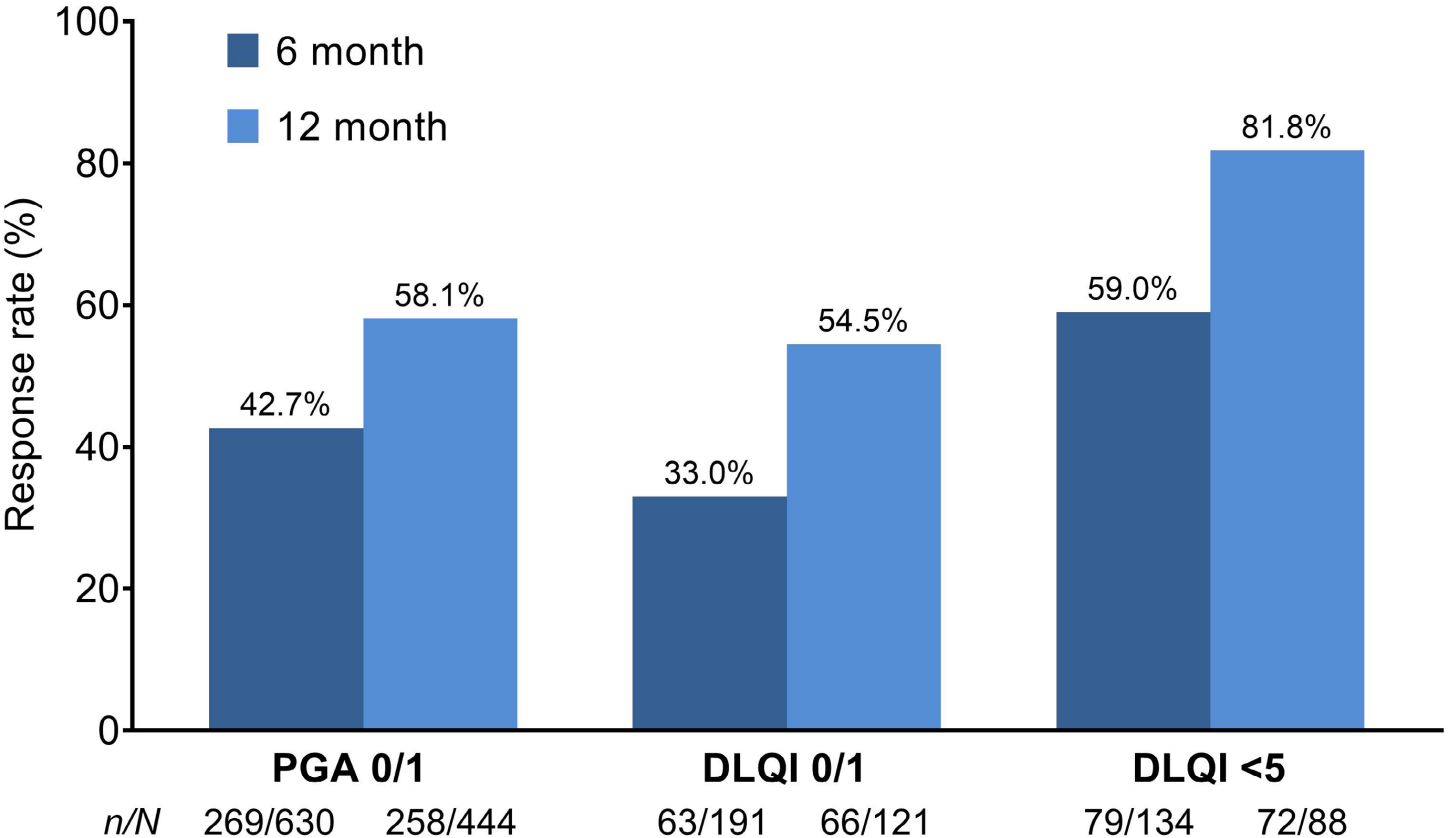
Response rate for Physician's Global Assessment (PGA) 0/1, Dermatology Life Quality Index (DLQI) 0/1 and DLQI <5 at 6 and 12 months in patients with plaque psoriasis (PsO). *n* indicates the number of patients assessed by PGA using 6‐ or 12‐month data from patients in whom each score could be calculated at two time‐points, at the start of apremilast treatment and 6 or 12 months after the start of apremilast treatment and whose PGA score at the start of treatment was not “None,” “Minor,” or “Non‐judgeable.” *N* indicates the number of patients assessed by total DLQI scores using 6‐ or 12‐month data from patients in whom each score could be calculated at two time‐points, at the start of apremilast treatment and 6 or 12 months after the start of apremilast treatment. SD, standard deviation.

The effectiveness rate in PsO was 90.9% after 6 months of apremilast treatment (Table 6) and 93.8% after 12 months (Table 7). The PGA score decreased significantly from baseline with a mean (SD) decrease of 1.4 (1.0) at 6 months and 1.6 (1.0) at 12 months (Tables 6 and 7; *p* < 0.0001 at both time‐points). A PGA score of 0/1 was achieved by 269 of 630 patients (42.7%) and by 258 of 444 patients (58.1%) after 6 and 12 months of treatment, respectively (Figure 3).

**TABLE 6:** Effectiveness of apremilast 6 months after treatment initiation in patients with psoriasis in the effectiveness analysis set.

	Baseline	At 6 months	*p*
Global improvement, *n* (%)		*N* = 747	
Highly effective		194 (26.0)	
Effective		485 (64.9)	
No effect		54 (7.2)	
Worsened		7 (0.9)	
Non‐judgeable		7 (0.9)	
Effectiveness rate (i.e., highly effective or effective)		679 (90.9)	
PGA score	*N* = 647	*N* = 647	
Mean (SD)	3.1 (0.8)	1.7 (0.9)	
Change in PGA score			
Mean (SD)		1.4 (1.0)	<0.0001
DLQI score	*N* = 212	*N* = 212	
Mean (SD)	7.7 (6.1)	3.5 (3.8)	
Change in DLQI score			
Mean (SD)	–	4.2 (5.4)	<0.0001

*Note*: Physician Global Assessment (PGA) and Dermatology Life Quality Index (DLQI) scores were calculated in patients in whom these scores could be calculated at the start and 6 months after the start of apremilast treatment.

Abbreviation: SD, standard deviation.

**TABLE 7:** Effectiveness of apremilast 12 months after treatment initiation in patients with psoriasis in the effectiveness analysis set.

	Baseline	At 12 months	*p*
Global improvement, *n* (%)		*N* = 518	
Highly effective		168 (32.4)	
Effective		318 (61.4)	
No effect		22 (4.2)	
Worsened		9 (1.7)	
Non‐judgeable		1 (0.2)	
Effectiveness rate (i.e., highly effective or effective)		486 (93.8)	
PGA score	*N* = 458	*N* = 458	
Mean (SD)	3.1 (0.8)	1.5 (0.9)	
Change in PGA score			
Mean (SD)		1.6 (1.0)	<0.0001
DLQI score	*N* = 137	*N* = 137	
Mean (SD)	7.9 (6.4)	2.1 (3.1)	
Change in DLQI score			
Mean (SD)	–	5.7 (6.8)	<0.0001

*Note*: Physician Global Assessment (PGA) and Dermatology Life Quality Index (DLQI) scores were calculated in patients in whom these scores could be calculated at the start and 12 months after the start of apremilast treatment.

Abbreviation: SD, standard deviation.

Improvements in quality of life were also observed; the total DLQI score decreased significantly from baseline (mean [SD] decrease of 4.2 [5.4] at 6 months; 5.7 [6.8] at 12 months; *p* < 0.0001 at both time‐points) (Tables 6 and 7). Following 6 months of treatment, 79 of 134 patients (59.0%) achieved a DLQI score < 5, with 63 of 191 patients (33.0%) achieving a DLQI score 0/1. Following 12 months of treatment, 72 of 88 patients (81.8%) achieved a DLQI score < 5, with 66 of 121 patients (54.5%) achieving a DLQI score 0/1 (Figure 3).

#### 3.3.2 Patients with PsA

**FIGURE 4:**
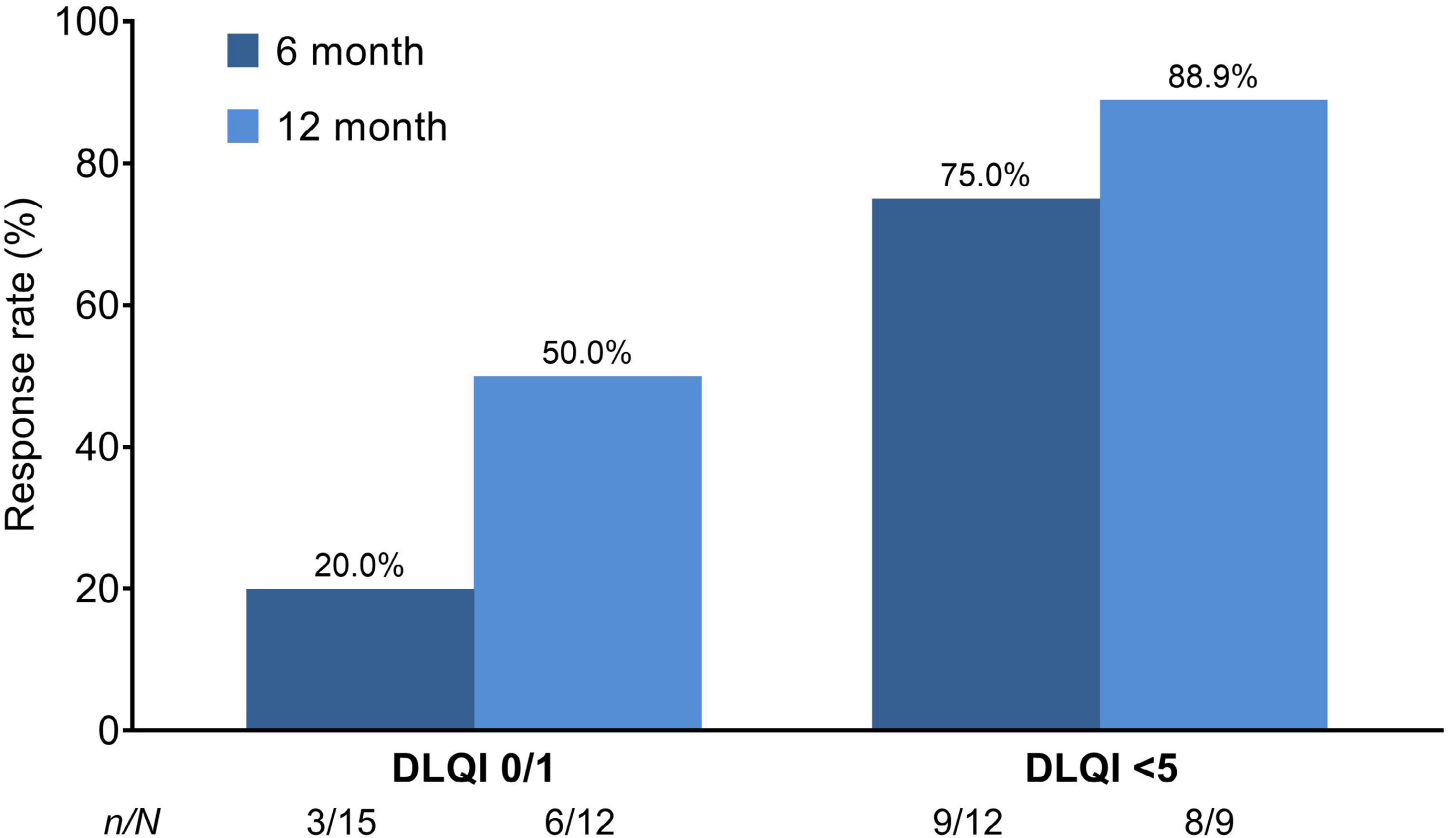
Response rate for Dermatology Life Quality Index (DLQI) 0/1 and DLQI <5 at 6 and 12 months in patients with psoriatic arthritis. *n* indicates the number of patients assessed by Physician Global Assessment (PGA) using 6‐ or 12‐month data from patients in whom each score could be calculated at two time‐points, at the start of apremilast treatment and 6 or 12 months after the start of apremilast treatment and whose PGA score at the start of treatment was not “None,” “Minor,” or “Non‐judgeable.” *N* indicates the number of patients assessed by total DLQI scores using 6‐ or 12‐month data from patients in whom each score could be calculated at two time‐points, at the start of apremilast treatment and 6 or 12 months after the start of apremilast treatment. SD, standard deviation.

At the 6‐month time‐point, 86 patients with PsA were included in the analysis for global improvement. At the 12‐month time‐point, this number decreased to 59 patients. The effectiveness rate of apremilast treatment was 88.4% after 6 months and 91.5% after 12 months. These results can be found in Tables S2 and S3. Following 6 months of treatment, nine of 12 patients (75.0%) achieved a DLQI score <5, with three of 15 patients (20.0%) achieving a DLQI score 0/1. Following 12 months of treatment, eight of nine patients (88.9%) achieved a DLQI score < 5, with six of 12 patients (50.0%) achieving a DLQI score 0/1 (Figure 4).

The VAS score decreased significantly from baseline (mean [SD] decrease of 25.2 [28.4] at 6 months [*p* < 0.0001] and 35.5 [26.2] at 12 months [*p* = 0.0001]). Similarly, the DAS28 score decreased significantly from baseline (mean [SD] decrease of 1.6 [1.1] at 6 months and 2.1 [0.7] at 12 months [*p* < 0.0001 at both time points]). After 6 and 12 months of apremilast treatment, remission was achieved by 52.6% and 77.8% of patients respectively, and a low disease activity was achieved by 21.1% and 22.2% of patients, respectively. The total DLQI score decreased significantly from baseline (mean [SD] decrease of 4.7 [4.1] at 6 months and 4.8 [4.5] at 12 months).

## 4 DISCUSSION

Results of this large‐scale, real‐world, PMS study in Japanese patients with PsO and/or PsA suggests that apremilast has an acceptable safety and tolerability profile and has positive clinical assessments of effectiveness and physician/patient‐reported outcomes. These safety and effectiveness results are generally consistent with findings of previously published international and Japanese phase 2b and 3 clinical trials.^5^, ^6^, ^7^, ^8^, ^9^, ^17^ Our results are also in line with those of three single‐center, real‐world studies in Japanese patients with PsO, conducted at Jichi Medical University,^18^, ^19^, ^20^ Saruwatari Dermatology Clinic,^21^ and Kurume University Hospital.^22^

Regarding safety, the incidence of ARs in this PMS study (312/1063, 29.4%) is similar to ARs determined in a phase 2b study in Japanese patients (71/241, 29.5%),^11^ but lower than the incidence of ARs calculated in a pooled analysis of international phase 2 and 3 studies (1046/2357, 44.4%).^4^, ^5^, ^6^ Diarrhea, nausea, and headache were observed as ARs in this PMS study, and as frequently reported AEs in previous international and Japanese clinical and real‐world studies.^4^, ^5^, ^6^, ^7^, ^8^, ^9^, ^10^, ^11^, ^17^, ^21^, ^22^, ^23^ The incidence of gastrointestinal ARs was higher in patients with a history of smoking, which represents one of the important findings of this study. It was also higher in patients with comorbidities as well as in those who had received prior and concomitant medications with no significant association between explanatory variables (such as sex, age, diagnosis, disease duration, or comorbidities) and the occurrence of gastrointestinal disorders as determined by multivariate logistic regression analyses.

Upper respiratory tract infections and nasopharyngitis were also frequently observed in clinical and observational studies of apremilast but were not commonly observed as an AR in this PMS study, suggesting a low possible causal connection with apremilast. Moreover, no serious infections were reported with apremilast in this study. In contrast, some biologics and Janus kinase inhibitors used in managing psoriatic disease in Japan may be associated with a higher risk of infections, which requires appropriate screening and monitoring of patients according to recommendations from Japanese guidance.^24^, ^25^

Overall, the rates of serious ARs remained low in this study and occurred only in 0.7% (7/1063) of patients, whereas the phase 2b study in Japanese patients reported an AR incidence of 2.4% (2/85 patients),^26^ and PROMINENT, a phase 3b study in Japanese patients with mild to moderate PsO,^17^ reported a similar incidence (2.6%; 4/152 patients) of serious treatment emergent adverse events (TEAEs).

PsO and PsA are chronic, systemic diseases associated with increased risk of cardiovascular and thrombotic events.^27^ In this PMS study, the occurrence of cardiovascular ARs with apremilast was low. This is consistent with the results of the pooled analysis of 15 clinical studies of apremilast that demonstrated a low incidence of major adverse cardiovascular events; they were nearly identical between apremilast and placebo groups in the placebo‐controlled period (0.1%).^23^

The incidences of ARs with apremilast related to the key survey items listed in this PMS study were low for weight decrease, malignancies, depression and suicidal events, and none of these events showed an increasing trend over time. Importantly, no instances of serious hypersensitivity, vasculitis or fatal ARs were observed in this PMS, further confirming that no new safety signals were identified.

In terms of onset, most ARs reported in this PMS, such as gastrointestinal disorders, were reported within 1 month of starting apremilast and gradually decreased over time. Consistent with this finding, AEs were typically reported in the first 2 weeks of apremilast treatment and resolved within 30 days throughout the ESTEEM and PALACE clinical trial programs in patients with PsO and PsA, respectively.^5^, ^6^, ^7^, ^8^, ^9^, ^10^ Resolution within 1 month was also typical for the gastrointestinal disorders of nausea and diarrhea, although these continued in some patients, highlighting the need for management. When ARs do emerge, it is important for physicians to manage these reactions by using shared decision making with patients,^28^ including expectations regarding management, likely resolution, and the importance of continuing apremilast as a systemic treatment, given the nature of PsO and PsA.

Apremilast demonstrated consistent improvement in disease severity measures and a patient‐reported measure of quality of life (DLQI) in this study, generally in line with the results of previously reported studies.^5^, ^6^, ^7^, ^8^, ^9^, ^10^, ^11^ Apremilast showed high effectiveness in this study, with global improvement seen in >90% of patients with PsO as well as those with PsA. Patients with PsO achieved a significant improvement in the severity of lesions and QoL. Patients with PsA achieved significant improvements in global improvement, joint pain VAS, DAS28, and DLQI; however, the effectiveness of apremilast in PsA in this study should be interpreted cautiously as the evaluable population for VAS, DAS28, and DLQI assessments was small.

Effectiveness results in terms of PGA 0/1 achievement was comparable between patients with PsO in this study and the efficacy results in the phase 3 PROMINENT study in Japan.^17^ Specifically, PGA 0/1 was achieved in 42.7% of patients in this study at 6 months compared with 43.7% of patients at 16 weeks in PROMINENT.^17^ In terms of quality of life DLQI 0/1 was achieved in 33.0% of patients at 6 months in this study and 41.2% of patients at 16 weeks in PROMINENT.^17^ The proportion of patients achieving DLQI 0/1 in our study at 12 months was higher than in a single‐center clinic study in Japan (54.5% vs 28.6%).^21^ In this study, the 12‐month survival rate was 46.8% whereas data from a retrospective study showed that the 12‐month survival rate of apremilast was 53.4% in Japanese patients with PsO or PsA.^19^

An increase in prescription of apremilast among oral PsO drugs (mainly because of its improved safety profile) was shown in a single‐center study^29^ and a similar trend was also reported in an epidemiological survey of PsO patients in the Japanese Society for Psoriasis Research.^30^ Apremilast, with its convenient oral administration and anti‐inflammatory effect but not immunosuppressive mode of action, favorable safety profile, as well as no requirement for laboratory monitoring, offers a unique therapeutic option for patients with PsO and/or PsA.^2^

Our study has several limitations including the non‐comparative open‐label, non‐randomized study design. This could result in selection bias, information bias, and attrition bias, all of which could affect the study results. It should also be noted that previous clinical and observational studies typically reported TEAEs rather than ARs (events reported by physicians other than those assessed as “not related” by physicians [including unknown/not specified]), as assessed in the current study. This makes direct comparison of results with previous studies difficult. Finally, the number of patients with PsA was relatively small, making the results in this population more difficult to generalize compared with results associated with patients with PsO.

In conclusion, this large, observational, PMS study demonstrated that orally administered apremilast was not associated with new safety signals and is well tolerated and effective in Japanese patients with PsO and/or PsA.

## FUNDING INFORMATION

Amgen K.K. conducted this study based on instructions from the Pharmaceutical and Medical Devices Agency, therefore, no funds or grants were received.

## CONFLICT OF INTEREST STATEMENT

Akimichi Morita has received research grants, consultancy fees, and/or speaker's fees from AbbVie, Amgen, Boehringer‐Ingelheim, Bristol‐Myers Squibb, Eisai, Eli Lilly Japan, Janssen Pharmaceutical, Kyowa Kirin, LEO Pharma, Maruho, Minophagen Pharmaceutical, Mitsubishi Tanabe Pharma, Nippon Kayaku, Novartis, Pfizer Japan, Sun Pharma Japan, Taiho Pharmaceutical, Torii Pharmaceutical, UCB Japan, and Ushio. Hidehisa Saeki has received research grants, consultancy fees, and/or speaker's fees from Amgen, Mitsubishi Tanabe Pharma, Taiho Pharmaceutica, Torii Pharmaceutical, Maruho, Kyowa Kirin, AbbVie, Novartis Pharma, Eli Lilly Japan, Eisai, LEO Pharma, Celgene, Janssen Pharmaceutical, UCB Japan, and Sun Pharma Japan. All other authors have no conflicts of interest to declare.

Hidehisa Saeki and Shinichi Imafuku are Editorial Board members of Journal of Dermatology and co‐authors of this article. To minimize bias, they were excluded from all editorial decision‐making related to the acceptance of this article for publication.

## Supporting information


Figure S1.

Table S1.

Table S2.

Table S3.

## Data Availability

The datasets used and/or analyzed during the current study are not available.
