# Supplementary material for: Safety and effectiveness of apremilast in Japanese patients with psoriatic disease: Results of a post‐marketing surveillance study
Source: J Dermatol. 2024 May 22;51(7):950–63. doi: 10.1111/1346-8138.17270 (PMC11484125; doi:10.1111/1346-8138.17270)
Supplement: Supplementary file 1 — Figure S1. [file JDE-51--s004.docx]

## Figure S1.

Patient disposition.


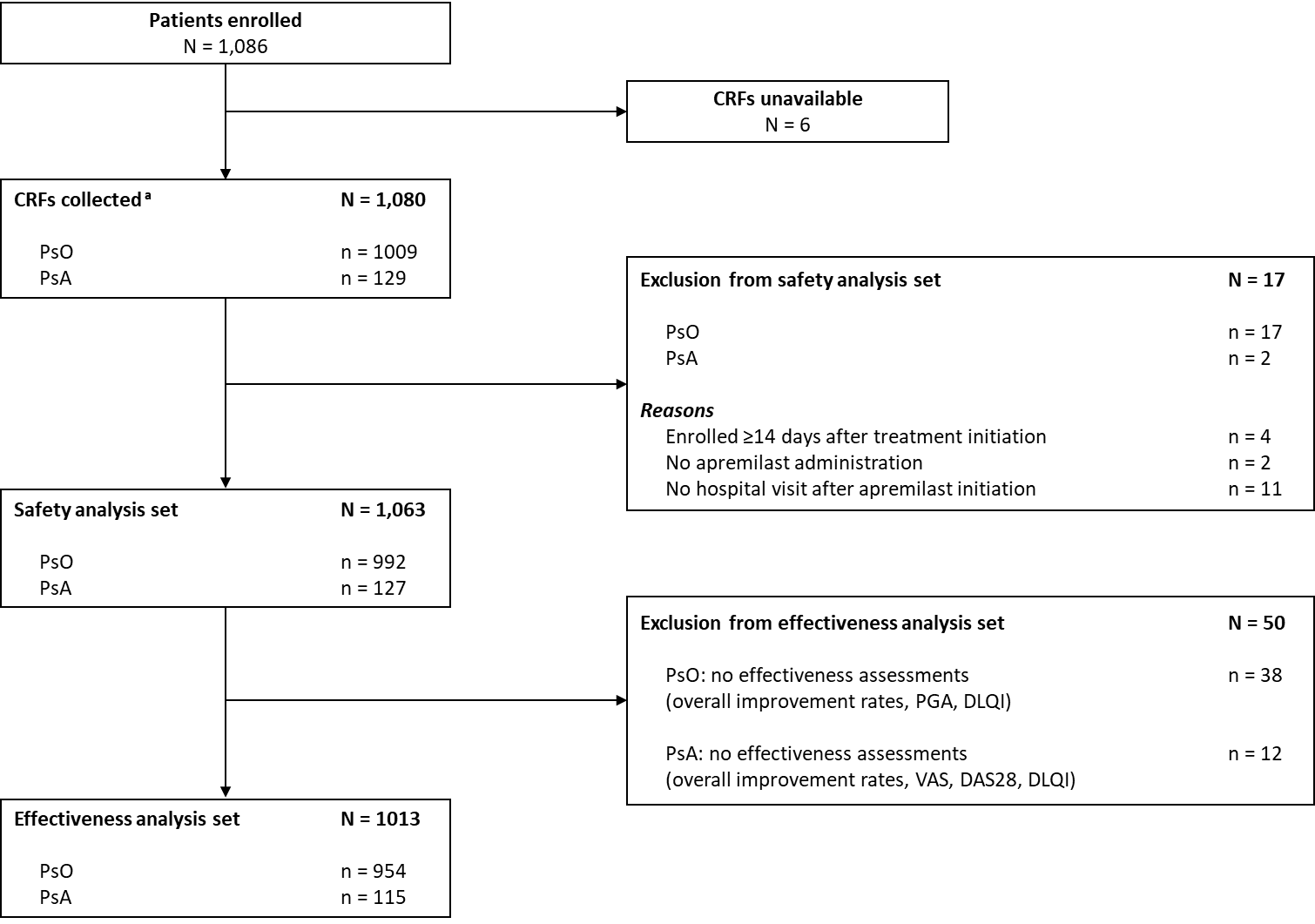


^a^Patients were counted once for each diagnosis (PsO or PsA) if they had more than one diagnoses.

PsA, psoriatic arthritis; PsO, plaque psoriasis.
