# Supplementary material for: Safety and effectiveness of apremilast in Japanese patients with psoriatic disease: Results of a post‐marketing surveillance study
Source: J Dermatol. 2024 May 22;51(7):950–63. doi: 10.1111/1346-8138.17270 (PMC11484125; doi:10.1111/1346-8138.17270)
Supplement: Supplementary file 2 — Table S1. [file JDE-51--s001.docx]

## Table S1.

**Apremilast dose and duration of treatment in the safety analysis set**

|  | **Overall**  **N=1,063** | **PsO**  **N=992** | **PsA**  **N=127** |
| --- | --- | --- | --- |
| Initial apremilast dose  (total daily dose), n (%) |  |  |  |
| 10 mg | 1046 (98.4) | 975 (98.3) | 126 (99.2) |
| 20–50 mg | 11 (1.0) | 11 (1.1) | 0 |
| 60 mg | 6 (0.6) | 6 (0.6) | 1 (0.8) |
| Total apremilast dose, mg |  |  |  |
| Mean (SD) | 14011.7 (8224.5) | 14023.3 (8222.0) | 13881.7 (8108.9) |
| Median (range) | 16260.0  (10.0–39330.0) | 16140.0  (10.0–39330.0) | 16290.0  (60.0–25410.0) |
| Duration of treatment, days |  |  |  |
| Mean (SD) | 253.5 (139.2) | 254.1 (139.0) | 248.7 (139.3) |
| Median (range) | 365.0 (1–658) | 365.0 (1–658) | 365.0 (3–426) |

PsA, psoriatic arthritis; PsO, plaque psoriasis; SD, standard deviation.
