# Supplementary material for: Safety and effectiveness of apremilast in Japanese patients with psoriatic disease: Results of a post‐marketing surveillance study
Source: J Dermatol. 2024 May 22;51(7):950–63. doi: 10.1111/1346-8138.17270 (PMC11484125; doi:10.1111/1346-8138.17270)
Supplement: Supplementary file 3 — Table S2. [file JDE-51--s002.docx]

## Table S2.

Effectiveness of apremilast 6 months after apremilast treatment initiation in patients with psoriatic arthritis in the effectiveness analysis set

|  | **Baseline** | **At 6 months** |
| --- | --- | --- |
| **Global improvement, n (%)** |  | N=86 |
| Highly effective |  | 19 (22.1) |
| Effective |  | 57 (66.3) |
| No effect |  | 6 (7.0) |
| Worsened |  | 4 (4.7) |
| Non-judgeable |  | 0 |
| Effectiveness rate (i.e., highly effective or effective) |  | 76 (88.4) |
| **VAS score** | N=27 | N=27 |
| Mean (SD) | 45.7 (25.5) | 20.6 (14.3) |
| Change in VAS score |  |  |
| Mean (SD) |  | 25.2 (28.4) |
| p-value |  | <0.0001 |
| **DAS28 score** | N=19 | N=19 |
| Mean (SD) | 3.7 (0.8) | 2.2 (0.7) |
| Change in DAS28 score |  |  |
| Mean (SD) |  | 1.6 (1.1) |
| p-value |  | <0.0001 |
| Disease activity assessment using DAS28 score, n (%) | N=19 | N=19 |
| Remission | 1 (5.3) | 10 (52.6) |
| Low disease activity | 0 | 4 (21.1) |
| Moderate disease activity | 11 (57.9) | 5 (26.3) |
| High disease activity | 7 (36.8) | 0 |
| **DLQI score** | N=17 | N=17 |
| Mean (SD) | 7.8 (5.2) | 3.1 (2.7) |
| Range) | 0–19.0 | 0–10.0 |
| Change in DLQI score |  |  |
| Mean (SD) | – | 4.7 (4.1) |
| Range | – | 0–12 |
| p-value | – | 0.0002 |
| DLQI 0/1 achievement rate, n (%) |  | N=15 |
| DLQI 0/1 | – | 3 (20.0) |
| DLQI <5 achievement rate, n (%) |  | N=12 |
| Patients with baseline DLQI ≥5 achieving DLQI <5 |  | 9 (75.0) |

DAS28, Disease Activity Score in 28 Joints; DLQI, Dermatology Life Quality Index; SD, standard deviation, VAS, Visual Analog Scale.

VAS, DAS28, and DLQI scores were calculated in patients in whom respective scores could be calculated at 2 time points, the start of apremilast treatment and 6 months after the start of apremilast treatment.
