# Supplementary material for: Safety and effectiveness of apremilast in Japanese patients with psoriatic disease: Results of a post‐marketing surveillance study
Source: J Dermatol. 2024 May 22;51(7):950–63. doi: 10.1111/1346-8138.17270 (PMC11484125; doi:10.1111/1346-8138.17270)
Supplement: Supplementary file 4 — Table S3. [file JDE-51--s003.docx]

## Table S3.

Effectiveness of apremilast 12 months after apremilast treatment initiation in patients with psoriatic arthritis in the effectiveness analysis set

|  | **Baseline** | **At 12 months** |
| --- | --- | --- |
| **Global improvement, n (%)** |  | N=59 |
| Highly effective |  | 13 (22.0) |
| Effective |  | 41 (69.5) |
| No effect |  | 3 (5.1) |
| Worsened |  | 1 (1.7) |
| Non-judgeable |  | 1 (1.7) |
| Effectiveness rate (i.e., highly effective or effective) |  | 54 (91.5) |
| **VAS score** | N=15 | N=15 |
| Mean (SD) | 47.2 (21.3) | 11.7 (15.3) |
| Change in VAS score |  |  |
| Mean (SD) |  | 35.5 (26.2) |
| p-value |  | 0.0001 |
| **DAS28 score** | N=9 | N=9 |
| Mean (SD) | 3.8 (0.6) | 1.7 (0.5) |
| Change in DAS28 score |  |  |
| Mean (SD) |  | 2.1 (0.7) |
| p-value |  | <0.0001 |
| Disease activity assessment using DAS28 score, n (%) |  |  |
| Remission | 0 | 7 (77.8) |
| Low disease activity | 0 | 2 (22.2) |
| Moderate disease activity | 6 (66.7) | 0 |
| High disease activity | 3 (33.3) | 0 |
| **DLQI score** | N=16 | N=16 |
| Mean (SD) | 6.4 (5.1) | 1.6 (1.7) |
| Change in DLQI score |  |  |
| Mean (SD) | – | 4.8 (4.5) |
| p-value | – | 0.0006 |
| DLQI 0/1 achievement rate, n (%) |  | N=12 |
| DLQI 0/1 | – | 6 (50.0) |
| DLQI <5 achievement rate, n (%) |  | N=9 |
| Patients with baseline DLQI ≥5 achieving DLQI <5 |  | 8 (88.9) |

DAS28, Disease Activity Score in 28 Joints; DLQI, Dermatology Life Quality Index; SD, standard deviation, VAS, Visual Analog Scale.

PGA and DLQI scores were calculated in patients in whom respective scores could be calculated at 2 time points, the start of apremilast treatment and 12 months after the start of apremilast treatment.
